# Facile synthesis of superhydrophobic surface of ZnO nanoflakes: chemical coating and UV-induced wettability conversion

**DOI:** 10.1186/1556-276X-7-216

**Published:** 2012-04-13

**Authors:** Lujun Yao, Maojun Zheng, Changli Li, Li Ma, Wenzhong Shen

**Affiliations:** 1 Laboratory of Condensed Matter Spectroscopy and Opto-Electronic Physics, and Key laboratory of Artificial Structures and Quantum Control (Ministry of Education), Department of Physics, Shanghai Jiao Tong University, Shanghai 200240, People's Republic of China; 2 School of Chemistry and Chemical Technology, Shanghai Jiao Tong University, Shanghai 200240, People's Republic of China

**Keywords:** ZnO nanoflakes, Chemical coating, Superhydrophobic, Corrosion protection, UV irradiation

## Abstract

This work reports an oriented growth process of two-dimensional (2D) ZnO nanoflakes on aluminum substrate through a low temperature hydrothermal technique and proposes the preliminary growth mechanism. A bionic superhydrophobic surface with excellent corrosion protection over a wide pH range in both acidic and alkaline solutions was constructed by a chemical coating treatment with stearic acid (SA) molecules on ZnO nanoflakes. It is found that the superhydrophobic surface of ZnO nanoflake arrays shows a maximum water contact angle (CA) of 157° and a low sliding angle of 8°, and it can be reversibly switched to its initial superhydrophilic state under ultraviolet (UV) irradiation, which is due to the UV-induced decomposition of the coated SA molecules. This study is significant for simple and inexpensive building of large-scale 2D ZnO nanoflake arrays with special wettability which can extend the applications of ZnO films to many other important fields.

## Background

Wettability of solid surfaces has been regarded as one of the most important morphology-dependent characteristics from both fundamental and practical viewpoints, and tremendous scientific interests are concentrated on functional surfaces with special wettability due to their excellent advantages over some particular fields. A superhydrophobic surface with a water contact angle (CA) greater than 150° and a water sliding angle less than 10° has been expected to inhibit snow sticking, contamination, erosion, and even current conduction [1-3]. While superhydrophilic surface with a water CA close to 0° has also prompted extensive interests such as fluid microchips [4] and papers in ink-jet printing [5]. Recently, with the development of smart devices, such as intelligent microfluidic switch and lab-on-chip systems, reversibly controlling the surface wettability has aroused great interest and been realized by chemical coating the surface with stimuli-responsive organic compounds. Various external inducement have been investigated to trigger this kind of conversion including ultraviolet (UV) light irradiation and dark storage [6,7], temperature [8], and electric field [9].

Being an important semiconductor, ZnO is a direct and wide bandgap (3.37 eV at room temperature), and it has been widely considered as a great electronic and photonic material used in UV photo detector, photocatalyst, gas sensors, solar cells, and others [10-17]. However, nearly all of the efforts are focused on the preparation of one-dimensional (1D) ZnO nanostructured arrays using kinds of approaches but few reports on the design of growing two-dimensional (2D) ZnO nanostructured arrays directly on special substrates [18,19] at low temperature and studies of their controllable wetting behavior.

In this paper, we report the oriented growth of 2D ZnO nanoflakes on bare aluminum substrate through low temperature hydrothermal route and reveal a detailed evolution of surface morphologies during the growth process. After surface coating with stearic acid (SA) monolayer molecules, the as-grown superhydrophilic surface of ZnO nanoflakes shows superhydrophobic property in the pH range from 2.3 to 12.1, which denotes that water contact angles are larger than 150° for not only pure water but also corrosive liquids, such as acidic and basic solutions, and the sliding angle is as low as about 8°. Also, we investigate the UV-induced chemical decomposition of the coated SA monolayer molecules on the ZnO nanoflake surface by means of X-ray photoelectron spectroscopy (XPS) analysis and CA measurement, and an opposite conversion from hydrophobicity to hydrophilicity is observed under UV irradiation. Therefore, the wettability of this kind of inorganic oxide films can be reversibly switched by alternation of UV irradiation and surface chemical coating with SA molecules.

## Methods

### Fabrication of oriented ZnO nanoflakes and chemical coating

Solution-based hydrothermal growth of 2D ZnO nanoflakes was achieved by dipping aluminum substrates in a capped Pyrex glass bottle filled with 16 mMol zinc nitrate hexahydrate (Zn(NO_3_)_2_·6H_2_O) and 16 mMol hexamethylenetetramine (HMT, C_6_H_12_N_4_); all chemicals were of reagent grade. The Pyrex glass bottle was sealed and maintained at a constant temperature of 90°C in a regular laboratory oven, and the reaction time was kept from 5-90 min to study the detailed growth process. Subsequently, aluminum substrates were taken out the solution, thoroughly washed with deionized water to eliminate any residual salts and dried by a nitrogen stream. SA molecules were chemisorbed on the ZnO nanoflake surfaces by immersing the sample (reaction time of 90 min) in an ethanol solution of 8 mMol SA (C_18_H_36_O_2_) for 24 h, followed by rinsing it in absolute ethanol to remove excess reactants, and then dried naturally.

### Analysis techniques and UV irradiation

Surface morphology was characterized by field emission scanning electron microscope (FE-SEM, Philips Sirion 200, Philips, Holland, The Netherlands). The X-ray diffraction (XRD) experiment was carried out with a D/max-2200/PC type diffraction, using CuKα radiation (λ = 1.5418 Å). Fourier transform infrared spectrum (FTIR) was measured by a spectrometer (Spectrum 100 FTIR, PerkinElmer, Waltham, MA, USA). An optical contact-angle meter system (Data Physics Instrument GmbH, Filderstadt, Germany) was used for static CA measurement at ambient temperature; liquid droplets of volume approximately 5 μl were suspended with needletube and brought in contact with ZnO nanoflake surface using a computer-controlled device. The sliding angle which reflects the relationship between advancing and receding contact angles was measured by tilting the sample platform of the optical contact-angle meter system until the water droplet rolled off the fixed sample. Surface chemical composition was analyzed by XPS (Kratos AXIS Ultra DLD, Shimadzu Corporation, Hadano, Kanagawa, Japan) at room temperature, the binding energies are calibrated with respect to the signal for adventitious carbon (284.8 eV).

To investigate the effect of UV irradiation on surface wettability of SA-coated ZnO nanoflake arrays, the sample was placed under the UV lamp (ZF-1 UV, Gu Cun, Shanghai, China), which emits UV light with a center wavelength of 254 nm, and light intensity was maintained at about 40 μW/cm^2^. Water contact angles and XPS peak intensities were recorded at different UV irradiation times at ambient temperature.

## Results and discussions

**Figure 1:**
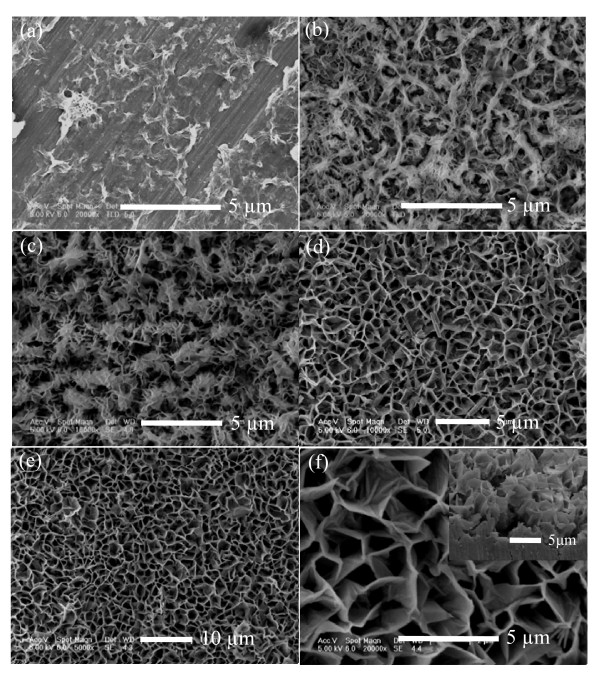
**FE-SEM images of the as-grown ZnO nanostructures on aluminum substrate in different stages**. FE-SEM images of the as-grown ZnO nanostructures on aluminum substrate in different stages, the reaction times are (**a**) 5 min, (**b**) 15 min, (**c**) 30 min, (**d**) 60 min, (**e**) 90 min. (**f**) A high magnification and cross-sectional images (shown in the inset) of 2D ZnO nanoflake arrays.

Figure 1 is the top view FE-SEM images of the as-grown ZnO nanostructures in different stages. When the reaction time is short (5 min), a few wrinkles can be observed on aluminum surface; however, the surface is very sparsely covered (Figure 1a). Upon increasing the reaction time to 15 min, these wrinkles become bigger and they are protuberant, as shown in Figure 1b. The color of aluminum surface changes from metallic sheen to a little gray indicating ZnO deposition. Figure 1c shows the FE-SEM image of the product with reaction time of 30 min, and we find out that when more and more ZnO nanoflakes have formed and self-assembled into clusters, aluminum substrate is partially covered with these clusters and ZnO nanoflakes are very small in width and height. A further increase in the reaction time (60 min) leads to an increased surface density of the clusters and nanoflakes, and when they begin to grow upwards and finally connect with each other (Figure 1d), it is found that each nanoflake is about 1.5 μm in width (defined as the horizontal size) and several tens of nanometers in wall thickness. Figures 1e, f are the low magnification and high magnification FE-SEM images of the as-grown ZnO nanoflakes. When the reaction time is 90 min, we clearly observe that these nanoflakes are 2 to approximately 3 μm wide, as the same size as their height, and they are nearly vertical to aluminum substrate, which was easily seen from the inset image of Figure 1f. Thus, an interesting continuous growth of 2D ZnO nanoflake arrays is achieved on the aluminum substrate.

**Figure 2:**
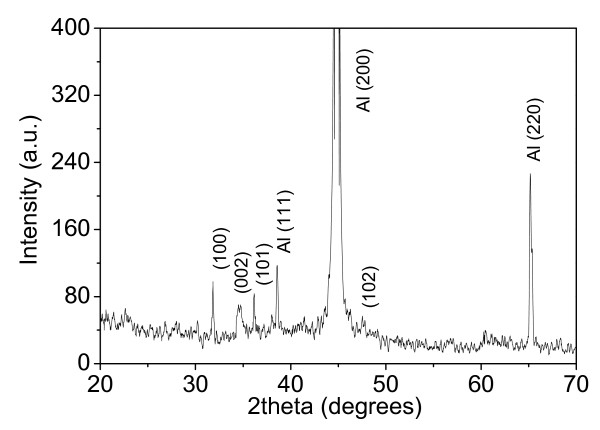
**XRD pattern of 2D ZnO nanoflake arrays revealing the wurtzite phase**.

Figure 2 is a representative XRD pattern recorded from the as-grown 2D ZnO nanoflakes. Three strong diffraction peaks are in good agreement with standard powder diffraction peaks of aluminum (Joint Committee on Powder Diffraction Standards (JCPDS) card number 85-1327), which originate from aluminum substrate. The other four weak diffraction peaks can be identified as hexagonal wurtzite phase of ZnO (JCPDS card number 76-0704). Previous studies have revealed that 1D ZnO nanorods will be produced if a silicon wafer, instead of aluminum, is used as the deposited substrate in the present experiment [9,20]. ZnO nanorods are synthesized by the reaction of Zn(NO_3_)_2_·6H_2_O and HMT, where HMT plays an active part in providing a controlled supply of OH^- ^anions by reacting with water [20]. The wurtzite ZnO crystal grows preferentially along [001] direction due to the lowest surface energy of (002) facet, and the growth velocity along [001] direction is the fastest leading to the formation of 1D nanorods [21]. As the substrate is aluminum, flake-like morphology of ZnO crystal grows, which is reasonable to presume that aluminum should be responsible for the suppression effect along [001] direction. Concerning the growth mechanism of 2D ZnO nanostructures, the (001) polar surface charges can be compensated by passivating agents [22,23]; a well-known example is the assistance of citric acid which absorbs preferably on the (001) surface of ZnO and then slows down the c-axis growth [23]. An increased OH^- ^concentration in solution can prevent new Zn(OH)_4_^2- ^ions from incorporating effectively and suppress the crystal growth along [001] direction [24]. In our experimental case, the passivating agent to control the growth of 2D ZnO nanoflake arrays should be Al(OH)_4_^- ^[25,26] which is formed by the chemical reaction between OH^- ^and aluminum substrate and presumably attaches to Zn^2+^-terminated (001) surface.

**Figure 3:**
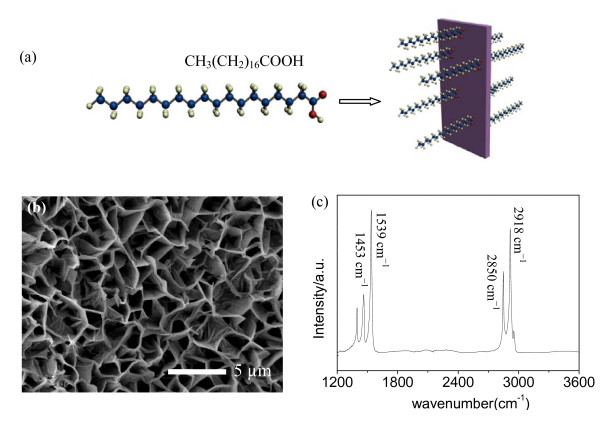
**Schematic model, top view FE-SEM image and FTIR spectrum of ZnO nanoflakes after chemisorptions**. (**a**) Schematic model of SA interaction with ZnO nanoflakes by grafting -COOH group onto the surface, (**b**)(**c**) top viewFE-SEM image and FTIR spectrum of ZnO nanoflakes after chemisorption of SA molecules.

Water CA measurement shows that the oriented ZnO nanoflake surface is superhydrophilic. When water droplets were dropped on the ZnO surface, they spread out rapidly in a few seconds and CA close to 0° is observed. This phenomenon was a lot different from some other reports [27-29] and in which ZnO nanostructured surfaces were often presented as superhydrophobic or partially hydrophobic properties, which could be attributed to their kinds of fabrication methods including plasma-enhanced chemical vapor deposition [27], spray pyrolysis [28], and thermal deposition [29] which was totally different from the low temperature hydrothermal technique. ZnO nanostructured films fabricated in high temperature often have lower defect density and lower surface free energies. In the other hand, aluminum substrate could also be an impact on the wettability because of Al(OH)_4_^- ^formation during the growth of ZnO nanoflakes. In order to tune surface wettability, we immerse the as-grown film in an ethanol solution of 8 mMol SA for 24 h. SA is known as a saturated flexible C18 hydrocarbon chain that stretches out in a long zigzag to form a dense self-assembled layer of packed chains on ZnO nanoflake surface as a result of the strong chelating bonds between carboxylic acid headgroups and Zn atoms on the surface; the schematic diagram is shown in Figure 3a. Figure 3b is the top view FE-SEM image of ZnO nanoflake arrays after chemical coating with SA molecules; there are no obvious morphological changes in comparison with the as-grown ZnO nanoflakes. SA molecules are absorbed onto ZnO nanoflake surface and lower its surface free energy [30]. The model of SA interaction with ZnO nanoflake arrays is confirmed by Fourier transform infrared spectroscopy (FTIR) spectrum (Figure 3c). Adsorption bands in accordance with the CH stretching mode peaks are clearly seen in the range of 2,800 to approximately 3,000 cm^-1^.

Two sharp peaks at about $2,918\;\text{c}{\text{m}}^{-1\;\nu_{a}}\left(\text{C}{\text{H}}_{3}\right)$ and $2,850\;\text{c}{\text{m}}^{-1\;\nu_{a}}\left(\text{C}{\text{H}}_{2}\right)$ indicate the existence of long-chain aliphatic groups and successful coating of SA molecules. The characteristic peak of the COOH group at 1,713 cm^-1 ^disappears after chemisorption, whereas two peaks appear at 1,453 cm^-1 ^and 1,539 cm^-1 ^were assigned to the symmetric and antisymmetric carboxylate ion COO^- ^stretching modes.

**Figure 4:**
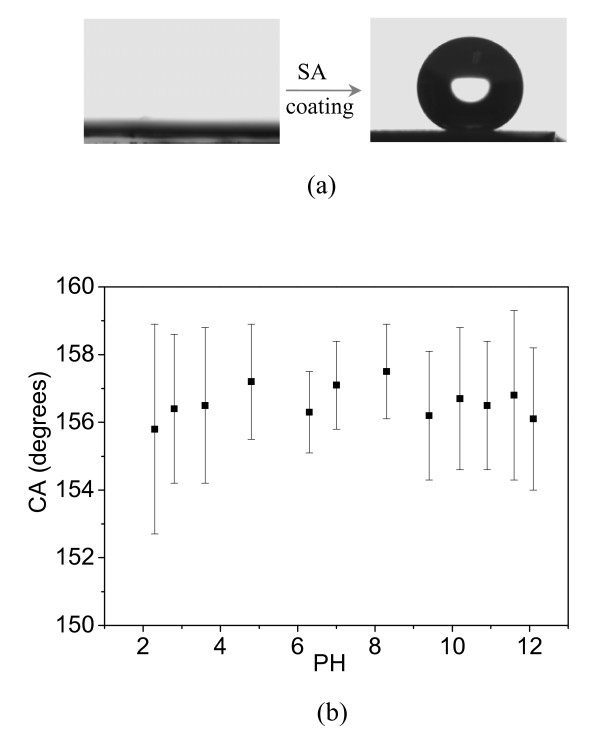
**Photographs of water droplet and contact angles of various buffered droplet**. (**a**) Photographs of a water droplet before and after chemisorption of SA molecules on the surface of ZnO nanoflakes, (**b**) contact angles of various buffered droplet (black squaresdenote the average CA values).

After surface chemical coating with SA molecules on ZnO nanoflakes, wettability can switch to superhydrophobic state (shown in Figure 4a), and the static water CA and sliding angle were measured to be about 157° and 8°, respectively. A combination of high surface roughness with low surface free energy obtained by SA chemisorption have been considered as the two key factors to superhydrophobic nature of this kind of SA-coated ZnO nanoflakes; a large fraction of air kept within the ZnO film can effectively increase the CA value according to Cassie model. Wetting stability is also evaluated by using approximately 5 μl droplets of buffered solution over a wide pH range (Figure 4b). It has been remarkable that ZnO nanomaterials is soluble in biofluids and dissolves in contact with mildly acidic or alkaline aqueous solutions [31]. But after chemical coating with SA molecules, the CA value of 2D ZnO nanoflake surface shows no apparent change within 3 min for an arbitrary solution when the pH is varied from 2.3 to 12.1, indicating a high stability and excellent corrosion protection from both acidic and alkaline solutions, This property can be explained that SA monolayer molecules act as an efficient barrier inhibiting buffered droplets to directly touch the ZnO nanoflake surface. CA values of each sample were measured for many times, and CA value at different PH was shown a different deviation form average CA value which was symboled as the black squares.

**Figure 5:**
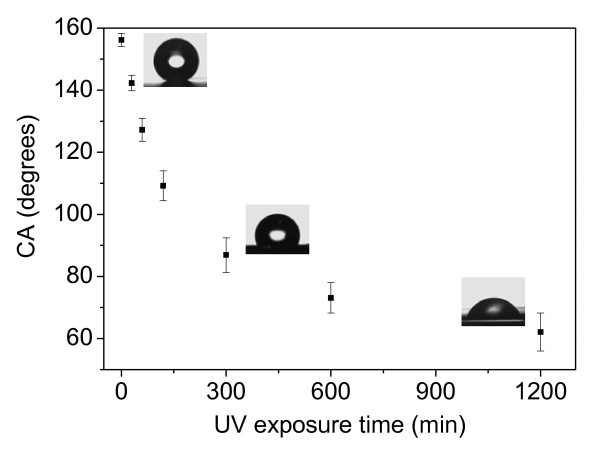
**Water contact angles as a function of UV irradiation time for the SA-coated ZnO nanoflakes**.

In order to study the influence of UV irradiation on surface-wettability response of the SA-coated ZnO nanoflake arrays, water contact angles are evaluated as a function of UV irradiation time in ambient conditions. A series of water contact angles between 157° to 60° are obtained which is dependent on the UV irradiation time, and the relationship is shown in Figure 5, black squares are the symbols of average CA values under different UV irradiation time and CA value was shown larger variation range with longer UV irradiation time, three representative photographs of water droplets corresponding to the UV irradiation time of 0 min, 5 h, and 20 h were inset, which are corresponding to superhydrophobic state, hydrophobic state, and hydrophilic state.

After UV irradiation over 5 h, a hydrophilic surface with CA less than 90° can be observed. When UV irradiation time is increased to 20 h, a water CA of about 61° shows. The wettability conversion indicates that UV irradiation efficiently decomposes the alkyl chains of SA on ZnO nanoflake surface in air. It has been revealed that alkylsiloxane monolayers can be slowly decomposed by OH radical and atomic oxygen made from UV dissociation of ozone, which is photogenerated from air [32]. A UV-induced decomposition mechanism of the self-assembled alky chains is also proposed based on the gas-phase oxidation mechanism of alkanes. OH radical and atomic oxygen firstly abstract hydrogen from alkyl chains and then produce alkyl radicals. Then alkyl radicals react to form alkoxy radicals, further producing reactive carbonyls through oxidation. Finally, these carbonyl groups dissociate through photodecomposition or attacked by radicals with the loss of carbon and thereby, gradually reduce the carbon chain length. Another possible decomposition mechanism is proposed on the photocatalytic effects [7]. As we all know, ZnO is a great semiconductor photocatalyst for organic compound degradation [7,11]. When ZnO nanoflakes are irradiated by UV light with photo energy higher than or equal to its bandgap, electrons in the valence band can be excited to the conduction band with the same amount of holes simultaneously generate in the valence band. The created holes and electrons will migrate to the surface of ZnO nanoflakes and initiate redox reactions with water and oxygen, leading to the decomposition of the alkyl chain of SA molecules in our experimental case. However, due to the weak UV light intensity (40 μW/cm^2^), CA can not reduce to 0° in a short time. If the UV irradiation time is increased to more than 80 h, a contact angle of less than 10° can be also acquired in this condition.

**Figure 6:**
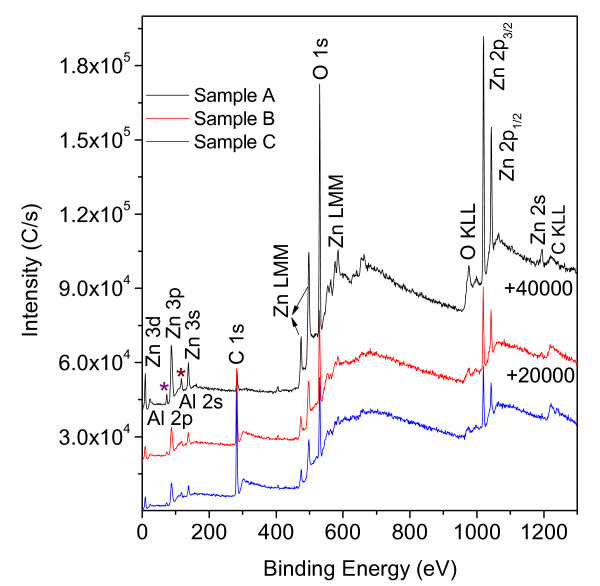
Wide scan XPS spectrum of the three different samples

**Figure 7:**
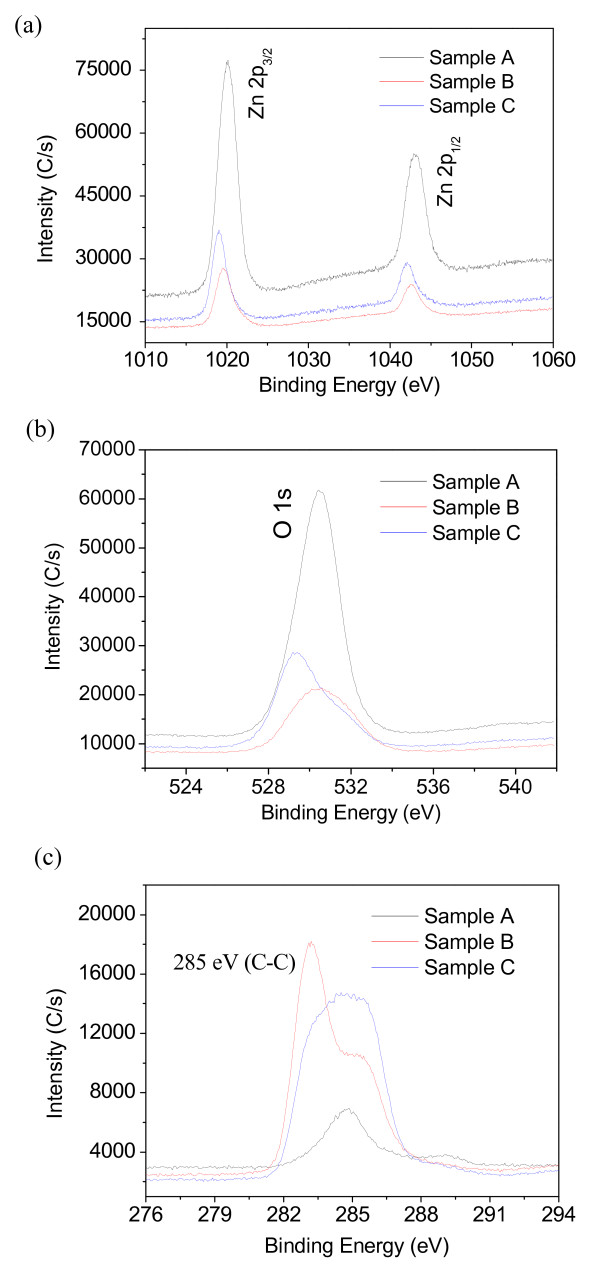
**Narrow-scan XPS spectrum of samples A, B and C**. Narrow-scan XPS spectrum of samples A, B and C taken from (**a**) Zn, (**b**) O and (**c**) C regions.

Surface chemical compositions have been characterized by means of XPS analysis in order to detect any trace of the photodecomposition of SA molecules; binding energies are calibrated with respect to the signal for adventitious carbon (284.8 eV). Figure 6 shows the wide scan XPS spectrum of as-grown ZnO nanoflakes (sample A), SA-coated ZnO nanoflakes (sample B), and SA-coated ZnO nanoflakes with UV irradiation time of 20 h (sample C). All the XPS peaks are similar and in agreement with the standard values of ZnO, but some peak intensities are different, such as Zn 2p_3/2 _(Zn 2p_1/2_), O 1 s and C 1 s. The detected Al 2p and Al 2 s peaks (denoted as 'asterisk') are from the aluminum substrates. A further investigation of the narrow-scan XPS spectrum recorded from Zn, O, and C regions are shown in Figure 7. It shows that Zn 2p_3/2 _(Zn 2p_1/2_) and O 1 s peak intensities of sample A are the strongest (Figure 7a), but for sample B, they are the weakest (Figure 7b). Figure 7c reveals the narrow scan of the C 1 s peak; it locates at about 285 eV which can be assigned to the C-C bond in the saturated carbon chains [33]. It is clearly seen that the C 1 s peak of the as-grown ZnO nanoflakes is weak. After chemical coating with SA molecules, the intensity of C 1 s peak becomes sharp and strong, revealing the successful coating of SA monolayer. However, the intensity of C 1 s peak decreases when the SA-coated ZnO sample is treated with UV irradiation time of 20 h, which indicates the decomposition of hydrocarbon chains and also the loss of well-packed SA monolayers.

The wide scan XPS spectrum of the as-grown ZnO nanoflakes (sample A), SA-coated ZnO nanoflakes (sample B), and SA-coated ZnO nanoflakes with UV irradiation of 20 h (sample C) (the peak intensities of samples A and B are added by 40,000 C/s and 20,000 C/s, respectively). *Asterisk *denotes the detected Al 2p and Al 2 s peaks from the aluminum substrates.

## Conclusions

In conclusion, we have demonstrated the oriented growth process of 2D ZnO nanoflakes on aluminum substrate through a low-temperature hydrothermal route; the growth mechanism was proposed on the basis of the Al(OH)_4_^- ^passivating agent formed by the chemical reaction between OH^- ^and aluminum substrate and then presumably attaches to Zn^2+^-terminated (001) surface. After chemical coating with SA monolayer molecules onto ZnO nanoflake arrays, surface wettability was converted from superhydrophilicity to superhydrophobicity with a maximum CA up to 157° and a low sliding angle close to 8°. This super water repellent surface revealed a stable property over a wide pH range; however, an opposite wettability conversion to hydrophilicity was observed under UV irradiation because of the cooperation of the surface photosensitivity and special chemical structure of SA molecules. This method possesses the advantages of being both simple and inexpensive, and special wettability of 2D ZnO nanoflakes can be reversibly switched by alternation of chemical coating and UV irradiation which is certainly significant for future industrial applications.

## Competing interests

The authors declare that they have no competing interests.

## Authors' contributions

LY participated in the design of the study, carried out the total experiments, performed the statistical analysis as well as drafted the manuscript. MZ participated in the design of the study, gave the theoretical and experimental guidance, performed the statistical analysis, and made the corrections of manuscript. CL mainly helped to carry out the measurement of CA and sliding angles. LM participated in the design of experimental section and supplied the help in experiments. WS helped to amend the manuscript. All authors read and approved the final manuscript.
